# Beyond Hot Spots: Biases in Antibody Somatic Hypermutation and Implications for Vaccine Design

**DOI:** 10.3389/fimmu.2018.01876

**Published:** 2018-08-14

**Authors:** Chaim A. Schramm, Daniel C. Douek

**Affiliations:** Vaccine Research Center, National Institute of Allergy and Infectious Diseases, NIH, Bethesda, MD, United States

**Keywords:** somatic hypermutation, hot spot motifs, affinity maturation, substitution profiles, vaccine design

## Abstract

The evolution of antibodies in an individual during an immune response by somatic hypermutation (SHM) is essential for the ability of the immune system to recognize and remove the diverse spectrum of antigens that may be encountered. These mutations are not produced at random; nucleotide motifs that result in increased or decreased rates of mutation were first reported in 1992. Newer models that estimate the propensity for mutation for every possible 5- or 7-nucleotide motif have emphasized the complexity of SHM targeting and suggested possible new hot spot motifs. Even with these fine-grained approaches, however, non-local context matters, and the mutations observed at a specific nucleotide motif varies between species and even by locus, gene segment, and position along the gene segment within a single species. An alternative method has been provided to further abstract away the molecular mechanisms underpinning SHM, prompted by evidence that certain stereotypical amino acid substitutions are favored at each position of a particular *V* gene. These “substitution profiles,” whether obtained from a single B cell lineage or an entire repertoire, offer a simplified approach to predict which substitutions will be well-tolerated and which will be disfavored, without the need to consider path-dependent effects from neighboring positions. However, this comes at the cost of merging the effects of two distinct biological processes, the generation of mutations, and the selection acting on those mutations. Since selection is contingent on the particular antigens an individual has been exposed to, this suggests that SHM may have evolved to prefer mutations that are most likely to be useful against pathogens that have co-evolved with us. Alternatively, the ability to select favorable mutations may be strongly limited by the biases of SHM targeting. In either scenario, the sequence space explored by SHM is significantly limited and this consequently has profound implications for the rational design of vaccine strategies.

## Introduction

In order to combat an arbitrarily large number of unknown pathogens, the humoral immune system relies on three mechanisms to generate diversity in antibody variable domains. In the primary repertoire, combinatorial diversity is created by the random joining of germline-encoded *V, D*, and *J* heavy chain or *V* and *J* light chain gene segments. During this process, junctional diversity is also introduced through the action of exonucleases and terminal deoxynucleotidyl transferase. This results in an estimated 10^15^–10^18^ possible unique naive B cell (1, 2). Furthermore, upon encountering cognate antigen, a naive B cell can enter a germinal center and begin to undergo somatic hypermutation (SHM), increasing the number of realizable antibodies by several additional orders of magnitude. However, the total number of circulating B cells in a human is only ~10^9^ (3, 4), meaning that if all possible antibodies were equally likely to be made, the odds of correctly producing one capable of binding to and clearing a particular antigen would be minuscule. In fact, precisely such arguments were initially used to argue against the “somatic” theory of antibody diversity predicting the existence of SHM (5). Hood and Talmage even pointed out that potential number of wasted mutations alone (i.e., those leading to non-functional antibodies and cell death) would far exceed the total number of cells thought to be produced over a human lifetime (6).

Nonetheless, the immune system has also evolved mechanisms for biasing the generation of diversity in ways, which presumably optimize the search for effective antibodies. For instance, different *V* gene segments are used at different frequencies (7, 8) and certain *D* genes may be more often recombined with specific *J* genes (9, 10). Many studies have shown that the parameters governing recombination vary dramatically from a uniform distribution and are generally reproducible between individuals (2, 11–14). Indeed, they appear to be optimized to produce B cells that can pass tolerance checkpoints and mature into naive B cells (2).

The SHM process is similarly biased. Soon after the first experimental confirmations of SHM (15, 16), it was quickly noted that mutations are more clustered together than random expectation (17) and fall into intrinsic hot spots (18, 19). Since the discovery of activation-induced cytidine deaminase (AID), the enzyme that initiates SHM by deaminating cytidine to uridine (20–22), much progress has been made in understanding the molecular origins of these biases. Many factors have been described that participate in targeting AID activity to the Ig loci by associating it with enhancer transcription and polymerase stalling [reviewed in Ref. (23–25)]. Studies of the specificity loop of AID (26–28) have elucidated the basis for the preferential deaminations of cytidines within specific microsequence motifs. Finally, investigations of uracil-DNA glycosylase, MutSα, DNA polymerase η, and many other components of the base excision and mismatch repair pathways have revealed some of the mechanisms behind patterns of mutations other than the C→T transitions generated directly by AID [reviewed in Ref. (25, 29, 30)].

The study of AID and other molecular components of the SHM machinery has always been complemented and even driven by computational approaches. For instance, the two-phase model of SHM (deamination by AID, followed by removal of the resulting uracil and error-prone repair) was first proposed in response to the observation that SHM is more focused on RGYW (where R is A or G; Y is C or T; and W is A or T) hot spots in MSH2-deficient mice (31). Similarly, the role of DNA polymerase η was deduced in part by comparing the motifs mutated by that enzyme to the WA hot spot motifs observed in SHM (32).

In addition, computational analysis can be clarifying, abstracting away molecular details to reveal higher level patterns such as the canonical RGYW hot spot motif itself. Recent work has suggested that the repertoire of nucleotide mutations generated by SHM can be further abstracted to amino acid substitution profiles (33, 34). These profiles point toward a new, simpler avenue for predictive analyses of the immune system, such as understanding potential responses to a specific vaccine immunogen. Here, we review the history, use, and limitations of microsequence motifs for predicting the targeting of SHM; the evidence that evolution has focused the SHM machinery toward producing specific types of amino acid changes at specific positions; the emerging use of substitution profiles and other similar predictive frameworks (FWR) for amino acid usages, along with their potential challenges and limitations; and how substitution profiles might find use in rational vaccine design.

## Microsequence Motifs

The idea that the diversity of antibody specificities could be attributed to ongoing accumulation of genetic mutations in proliferating lymphocytes was first proposed by Lederberg (35). Brenner and Milstein then suggested a mechanism based on DNA cleavage targeted to specific genetic loci, followed by exonuclease activity and error-prone repair (36). After the emergence of experimental support for this hypothesis (17, 37), analogy to the action of known mutagenic agents led Rogozin and Kolchanov to examine the possible influence of neighboring bases on the occurrences of mutations in antibodies. This resulted in the discovery of the now-canonical RGYW/WRCY hot spot motif (where the underline indicates the mutated base), as well as the apparently equally mutable TAA motif (38). Later, a disfavored cold spot motif of SYC (where S is C or G) was also reported (39).

However, despite the usefulness of the WRCY and TAA motifs, only about 30% of observed SHMs fall into such hot spots (38). Moreover, it quickly became clear that not all 8 WRCY sequences were equally “hot,” with AGCT being favored (19, 40–42) and AGCC or TGCG being disfavored (43, 44). At various times, WRCH (where H is A, C, or T) (45), WRCR (46), and WRCW (47) have been suggested as more accurate motifs, with the WRC thought to be the core motif (39, 46), with the last base possibly influencing the choice of repair pathways (45). Similarly, the originally proposed TAA motif was later refined to WA (32). In addition, other potential hot spot motifs have been suggested, such as CRCY and ATCT (48).

Another approach has been to explicitly calculate mutation rates for each possible nucleotide sequence of a given length. In the first such study, Smith estimated the relative mutability for all possible di- and trinucleotide motifs using downstream J_K_ sequences from mouse hybridoma lines, concluding that the dinucleotides explained most of the variation in mutational targeting (40). They later extended this analysis to mouse and human heavy chains (49) and human kappa chains (50), using non-productive rearrangements instead of intronic sequences to calculate mutabilities in humans (49, 50). They found broad similarity between species and between heavy and kappa (49, 50), while a later analysis of non-productive human IgL sequences with higher mutation levels suggested substantial differences from IgH (51). Ohm-Laursen used non-productive rearrangements of V_H_3-23 with J_H_4 or J_H_6 to derive a quartet model and showed that the frequency of mutation at specific motifs in the D and J genes correlated well with those in the V gene (43). A different quartet model used the V gene region of all publicly available antibody sequences and modeled the effects of the flanking nucleotides as independent from the position of the mutation itself (52). These authors found a high correlation of observed quartet mutation frequencies (~0.7) between heavy and light chains and between human and mouse antibodies. However, the full model could only explain around half of the variation in mutation frequencies in the real data (52).

More recently, with the advent of high-throughput sequencing technologies, attempts have been made to build out more finely discriminatory models. Yaari constructed a 5 nucleotide motif model using only synonymous mutations from functional sequences (44). The frequency at which each motif was targeted was highly correlated between individuals (~0.9), but the correlation between expected and observed mutations was only 0.67. Moreover, 46% of possible 5-mer motifs were not observed directly and had to be estimated from other similar motifs (44). The same group also immunized mice transgenic for the B1-8 heavy chain with (4-hydroxy-3-nitrophenyl)acetyl, which produces a response heavily biased toward λ chain usage (53). They sequenced the non-productive kappa chains from these animals and confirmed that the 5-mer mutation frequencies from functional and non-functional sequences correlated well with each other (48). They also built 5-mer models for mouse heavy chains and human light chains, finding an overall correlation of only 0.63 between the species. Specifically, C:G base pairs were observed to be more likely to mutate in mice and also to have a higher probability to result in a transition substitution than in humans (48).

To overcome the limitation of motifs that do not appear in the repertoire of germline Ig sequences, Elhanati et al. constructed a 7-nucleotide position weight matrix (PWM) that treats each position independently, finding a correlation of 0.8 between predicted and observed mutations frequencies (2). A later refinement of this model also calculated 7-mer PWMs for *D* and *J* gene-derived nucleotides, finding that those differed sharply from the PWMs learned for *V* genes (54). Another new approach, termed “samm,” uses a proportional hazards model with a lasso penalty and a flexible motif dictionary to extract the most important features and construct motifs accordingly (55). When used to build a 5-mer motif model and compared directly to Cui et al. (48), the results are similar, but samm tended to discount the effect of the final nucleotide, inferring only 382 unique mutability values instead of 1,015 (55).

In addition to calculating the frequency of mutations at each motif, many groups have investigated the resulting mutation spectrums, or the relative rates of mutation to each possible destination nucleotide. Although a preference for transitions over transversions was first reported in the early 1990s (19, 56), Cowell and Kepler were the first to report a dependency on neighboring bases for mutations spectrums (57). They found that both nucleotides in a homodimer have an increased propensity for transitions, while AT and TA dinucleotides have a preference to mutate to AA or TT homodimers (57). Ohm-Laursen calculated mutation spectrums for all 4-nucleotide motifs (43), but did not specifically analyze the effects of context. The quartet model of Cohen calculated mutation frequencies independently for each possible destination nucleotide, finding that the particular substitution had as much impact on the variability of mutation frequencies as did the microsequence context (52). Several other groups have calculated mutation spectrums, as well (2, 44, 48, 54) though those authors all deemphasized mutation spectrums compared to mutation frequencies or general properties of the antibody repertoire. This is due to the fact that mutation spectrums are considered less computationally tractable, as the underlying molecular machinery is significantly more complex and less well understood. In addition, they have been thought to be less useful, as the observed substitutions are presumed to be heavily influenced by selection for antigen binding, which acts on the amino acid sequence. One attempt has been made to parameterize an amino acid substitution matrix for antibodies (58), which does not compare favorably to real data when used to simulate SHM (33).

Even extended to 5- and 7-nucleotide motifs, microsequence context can only account for 70–80% of variability in mutation frequencies (2, 44, 52, 54). Much of the residual variation appears to be due to positional effects within the antibody sequence. Differences between FWR and complementarity determining regions (CDR) have been reported (49, 50, 52, 59), and regional variation can be observed even in non-Ig transgenes (59). In addition, mutation frequencies for the same sequence decay exponentially with distance from the transcription start site (60). In addition, differences between the heavy, kappa, and lambda chain loci are consistently observed (48, 49, 51, 52). The complex interdependence among all of these factors suggests that an evolutionary balancing has optimized the types and distributions of mutations produced by SHM.

## Evolutionary Optimization of SHM

One of the primary selective pressures driving antibody gene evolution is the need for functional diversity. Antibody genes were originally thought to be subject to “coincidental” or “concerted” evolution, as seen for other multigene families like ribosomal RNA and histone genes, with diversity generated by unequal crossing over and/or gene conversion (61, 62). However, an early study of the phylogenetic relationships between mouse and human V_H_ genes suggested that the rate of V_H_ gene duplication would have to be over 100-fold lower than for other multigene families (63). Later, studies with access to more sequences from more species were able to show that V gene evolution is instead governed by a “birth-and-death” process, which results in a more dynamic and diverse repertoire between species (64, 65). Within V_H_ genes, moreover, the germline sequences of the CDRs, but not FWRs, are under diversifying selection (63, 66). In addition, SHM is itself an evolutionarily ancient diversification mechanism, preceding the emergence of combinatorial *V*(*D*)*J* joining and the full diversification of V_H_ genes (67). SHM has been observed *in vivo* in the horn shark (67), and AID orthologs with *in vitro* deaminase activity have been isolated from cartilaginous fish (68) and even jawless vertebrates (69). Although all of the AID orthologs tested retained a general preference for WRC motifs over non-WRC substrates, the exact microsequence specificity varied substantially (68), suggesting co-evolution of the SHM machinery with antibody gene sequences to optimize the humoral immune response.

The interplay between evolution of the primary sequences of the germline repertoire and the biased mechanisms of SHM can also be seen in the fact that the codon composition of CDRs make them more prone to replacement mutations, while the structurally important FWRs use codons that are biased toward silent mutations (70–72). Similarly, Wagner et al. found that highly mutable AGY codons are preferentially used to encode serines in CDRs, while less mutable TCN codons tend to appear in FWRs (73). Kepler reported a general difference in codon usage between CDRs and FWRs, which was strongly correlated with differential mutability (74). Moreover, both the specific serine bias (75–77) and the general codon bias (78) appear to be phylogenetically conserved, emphasizing the importance of plasticity in the CDRs. In fact, recent work has demonstrated that AGC hot spot triplets in the CDRs are specifically conserved in the serine reading frame (79). These codons are exceptionally plastic, and mutated AGY serine codons are disproportionately involved in antigen contacts seen in crystal structures (79).

Shaping of the action of SHM extends beyond differences between CDRs and FWRs. For instance, Zheng et al. showed that C→T transitions are predominantly silent, and that those which would lead to replacement mutations are found primarily in cold spots (80). A similar, though less strict, distribution was reported for G→A transitions. Those authors speculate that this pattern might have evolved to keep mutations created directly by AID from overwhelming those caused by error-prone repair in phase II (80).

Somatic hypermutation is also targeted to be able to introduce gross structural changes to antibodies in a favorable way. For instance, mutations are frequently observed in human Vκ1-derived antibodies at two FWR positions, which affect interdomain dynamics and enhance thermostability (81). Similarly, sequences that can give rise to an NxS/T glycosylation motif with only one nucleotide change are concentrated in the antigen-proximal loops of the variable domains (82).

Evolution appears to shape the naive repertoire, as well. Recent work has demonstrated that observed biases in the usages of various V gene segments correlates with the predisposition of each gene to focus SHM toward its CDRs (72). More generally, the likelihood that the antibody encoded by an immature B cell can survive central tolerance and get selected into the naive repertoire correlates with the likelihood of that sequence being generated by the recombination machinery in the first place (2). In a similar vein, mouse antibodies have substantially less D_H_ gene variation and junctional diversity than humans, which has been hypothesized to overcome the limitations of a numerically small B cell population by focusing the naive repertoire on the most critical specificities (83).

Even in humans, these biases allow the development of stereotyped antibodies, specific recombinations using particular genetic elements that can be reproducibly elicited by a particular antigen (12). These stereotyped antibodies can even target complex antigens such as influenza HA (84–86) and HIV Env (87, 88). In addition to stereotyped genes, the antibody response can reproducibly make use of specific amino acid substitutions generated by SHM. This had been observed in both mice (18, 89) and humans (84, 85, 90), and even when the mutation in question occurs in a cold spot of AID activity (91). Moreover, substitutions that appeared in V_H_1-46-derived antibodies targeting the CD4-binding site of HIV Env from multiple donors were also observed in V_H_1-46-derived antibodies from HIV-uninfected donors (92). This demonstrates that shared substitutions can occur in the selected functional repertoire even without a common antigen and may reflect the way that the SHM machinery has evolved to sample the mutations that are most likely to be useful.

## Substitution Profiles

It seems counterintuitive that *a priori* predictions can be made about the state of the selected functional repertoire without reference to the antigens that have driven that selection. However, the number of unique clones in which a particular position has been substituted is correlated with the diversity of germline amino acids available at that position, in both CDRs and FWRs (93). Strikingly, the diversity of substitutions at changed positions is also correlated with germline diversity, though the diversity of the germline amino acids is less than that of the substitutions (93). While this at least partially reflects the structural constraints of the antibody domain, the physicochemical properties of the observed substitutions did not generally parallel those of the germline residues at the same positions (93).

**Figure 1:**
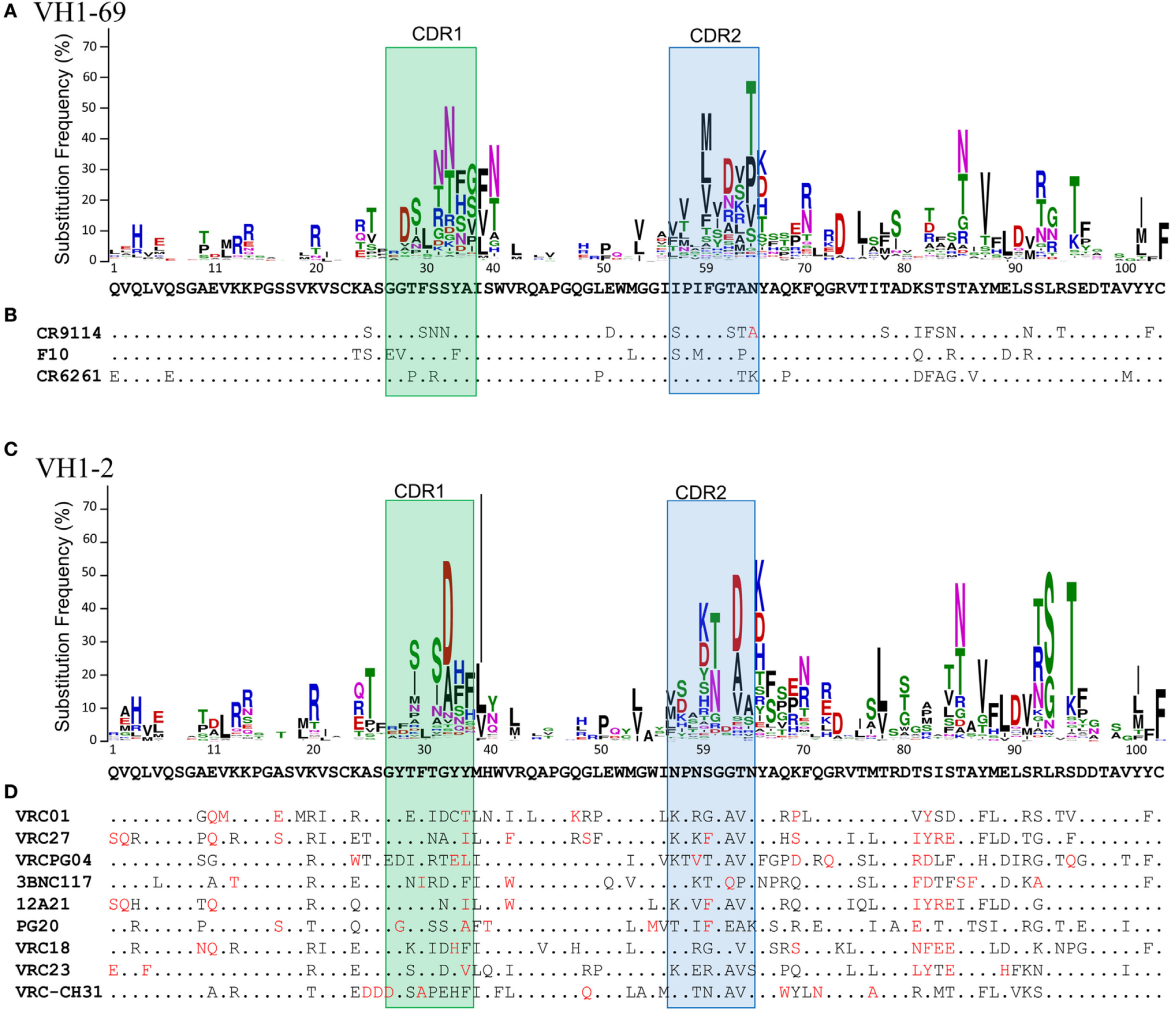
Substitution profiles and the accumulation of rare mutations. **(A)** Substitution profile of VH1-69 as calculated by Sheng et al. (33). The germline amino acid sequence is shown below the logo plot. **(B)** Sequence alignment of VH1-69-derived influenza antibodies capable of neutralizing multiple subtypes. Dots indicate the germline amino acid and extremely rare substitutions [as defined in Ref. (33)] are shown in red. **(C)** Substitution profile of VH1-2. **(D)** Sequence alignment of VH1-2-derived broadly neutralizing HIV antibodies targeting the CD4 binding site. Each contains greater than ~20% or more extremely rare substitutions.

In fact, the diversity of the observed substitutions is constrained not only by the diversity of all germline genes at a position but specifically by the particular gene from which the antibody was derived (33, 34) (Figure 1). As seen in studies of microsequence motifs (44) and *V*(*D*)*J* recombination (2, 11, 13, 91, 94), these substitution profiles are stable between individuals and across time (33). Similar findings have been reported both for sequences isolated from peripheral memory B cells (33) and from bone marrow cells (34). Both substitution frequency and the diversity of observed substitutions are generally higher in CDRs than in FWRs, though several FWR positions have substitution profiles similar to those characteristic of CDRs (33, 34). In addition, assorted V_H_ genes accumulate substitutions in CDRH1 versus CDRH2 at different rates, and similar variations appear in the preferred locations of insertions and deletions (34).

Many expected factors contribute to the observed substitution profiles. For instance, the frequencies of substitution are generally lower at structurally important residues such as the charge cluster (95), though individual genes may display higher rates, as for R95 of V_H_1-8 (34) [residue numberings are reported using the IMGT convention (96)]. Similarly, when a particular gene carries a residue that is distinct from other genes in its V_H_ family (e.g., L71 in V_H_1-18 and T46 of V_H_1-8), substitutions at that positions are frequently biased toward the germline residue(s) encoded by the other members of the gene’s family (in this case, F and P, respectively) (34). The presence or absence of a microsequence hot spot also clearly impacts the observed differential substitution rates at some positions, such as S29 in V_H_5-51, which forms an AGCT hot spot and diversifies extensively, while the same serine in V_H_4 genes is encoded by a TCC codon and mutates only rarely (34). However, it cannot account for all such differences; for instance, R80 in V_H_1-8 diversifies extensively despite the absence of a canonical hot spot, while the equivalent arginine in V_H_3 genes is almost complete conserved, without the presence of an AID cold spot (34). Simulations indicate that microsequence motifs can account for about 70% of the variation in substitution frequencies, similar to previous reports (44), but only about 50% of the variation when the identity of the substitutions is included (33). Another contributing factor to substitution profiles is the fact that observed substitutions are typically those that can be reached by a single nucleotide change. However, the same codon can have substantially different profiles even in a highly similar sequence context. Thus, the TCC codon encoding S83 of V_H_1-2 is most likely to be substituted to A, followed by T and P, while the most likely substitutions for S83 of V_H_1-46 are P and F, followed by A (33). Furthermore, while biases in substitutions are somewhat correlated with the physicochemical similarity between the germline amino acid and the observed substitution, many commonly seen substitutions are non-conservative, and even conservative substitutions are frequently asymmetric (e.g., E→D and K→R substitutions are more likely to occur than D→E and R→K substitutions, respectively) (33, 97).

In addition to highly significant similarities in substitution profiles between individuals with presumably distinct antigen-exposure histories, substitution profiles observed in the selected functional repertoire are also correlated to those derived from non-functional passenger alleles (33). This convergence of the selected and unselected repertoires is quite surprising and implies stricter limitations on the action of SHM than had previously been understood. One possibility is that the evolutionary optimizations described above are fine-tuned enough to strongly bias the production of mutations toward those that are most likely to be selected for by the suite of antigens that has been most commonly encountered over the evolutionary history of a species (33). In this vein, recent work has shown that relatively low-affinity antibody lineages can persist in germinal centers responding to complex protein antigens (98–100). This results in a memory response with increased clonal diversity compared to that generated by haptens, and it has been hypothesized that this diversity enhances the capacity of the immune system to respond to future challenges from novel but structurally related antigens (101). It may be that the characteristic substitutions observed in substitution profiles serve to optimize the structure to this diversity. An additional, perhaps complementary, alternative is that the biases in the mutations produced by the SHM machinery are strong enough that most mutations are not produced frequently enough to be acted upon by selection. In either case, there would appear to be drastic implications for rational vaccine design efforts, as certain substitutions may not be reliably available in a typical repertoire, even with an optimal antigen.

More generally, the existence of substitution profiles indicates that there are preferred pathways for antibody affinity maturation that depend powerfully on the germline gene used. This, in turn, suggests that germline-based substitution profiles contain useful information about which substitutions are likely to be tolerated at each position, which can be leveraged for antibody engineering. As most engineering efforts begin from a known monoclonal antibody, a narrower substitution profile, encompassing a single antibody lineage, may be of particular use (102). These lineage-specific substitution profiles are expected to be different from gene-specific substitution profiles (33), but may better reflect the constraints of binding to a specific antigen. They also provide an opportunity to extract information about which substitutions can be tolerated at positions in CDR3 and FWR4, which are absent in V gene-specific profiles. Frequently, however, the antibody that is being engineered is the only known member of its lineage; even when deep repertoire sampling is done with high-throughput sequencing, most lineages are represented by only one or a few members (103).

A new program named SPURF attempts to overcome that limitation by combining several types of substitution profiles derived from a large public data set to predict the substitution profile of an antibody lineage from the sequence of a single member (102). In training the SPURF model, the authors found that the most important sources of information are the *V* gene-specific substitution profile and the inferred naive sequence, in addition to the input sequence itself. They also use a gene-family substitution profile (i.e., derived from all V_H_1 genes, etc.) and a substitution profile calculated from simulations of neutral mutation of the inferred naive sequence using the S5F model from reference (44, 102). In particular, the inclusion of the inferred naive sequence allows the prediction of a substitution profile for CDR3 and FWR4, which are not encoded by the *V* gene and, therefore, missed by a *V* gene-specific profile alone.

## Open Questions

While SPURF performs well predicting the lineage-specific substitution profiles of an out-of-sample validation set (102) and is designed to be used for antibody engineering and improvement, it has not yet been tested in that context. Similarly, it remains to be seen if substitution profiles can be successfully incorporated into a predictive model of SHM. And while rare substitutions can be functionally important (104), systematic comparisons of the structural and biophysical effects of common versus rare substitutions are ongoing. In addition, substitution profiles treat the mutations observed at each position as being independent. However, recent work suggests that affinity-enhancing mutations may be co-selected with structurally stabilizing ones (105), and the possibility of correlations between the substitution profiles of different positions should be investigated.

Another open question involves the effects of allelic variants on substitution profiles. Even silent polymorphisms could theoretically change the pattern of mutations generated by SHM by the introduction or removal of a microsequence hot spot. More importantly, allelic variants are sometimes distinguished by replacement mutations (e.g., G55 versus R55 in V_H_1-69). Since the germline residue remains the most commonly observed amino acid at most positions, these variants will have a large impact on the resulting substitution profile. So far, this has been handled in an *ad hoc* manner, by either excluding genes from donors who have previously been determined to be heterozygous for such variants (34) or by collectively excluding all possible germline residues at each position from the substitution profile, irrespective of individual genotype (33). Since the germline residues at homologous positions in closely related genes are frequently observed substitutions (34), a more systematic way of investigating the effects of allelic variants is necessary. This is especially true as it has recently become clear that many such variants remain to be discovered (106–109).

Finally, one of the most striking findings about substitution profiles is the similarity of the selected and unselected repertoires. Yet, this observation rests on mere 650 non-productive rearrangements derived from a single V_H_–J_H_ gene pair (33, 110). Although the strong correlations between substitution profiles from different individuals also support the idea that SHM is capable of generating only a limited set of mutations, more data are needed to test this. Meanwhile, it is clear that mutation and selection are distinct biological processes. In order to avoid possible confounding effects of selection, studies of microsequence motifs have typically used sequences derived from introns, transgenes, or non-productive rearrangements; or, if using sequences from functional antibodies subject to selection, have included only silent mutations extracted from those data sets.

Separately, many efforts have been made to detect and quantify the action of selection on affinity maturation. Initially, these evaluated the frequency of replacement mutations observed in CDRs versus FWRs using a binomial (111) or multinomial (112) distribution. The binomial model has also been extended to account for codon biases that lead to a higher neutral rate of replacement mutations CDRs (70) and to account for general differences in mutability driven by microsequence context (113). However, determining the appropriate null distribution of replacement versus silent mutations in antibodies has proven challenging, as the intrinsic biases of SHM can give the appearance of selection (114) even when microsequence motifs are accounted for (113). One strategy for addressing this difficulty has been to use a focused binomial test examining the replacement mutations from only a single CDR or FWR at time, while using the silent mutations from all regions (115, 116). Another strategy exploited a large data set of non-productive rearrangements to normalize the ratio of replacement to silent mutations on a germline- and position-specific basis (94). Other recent advancements include the use of a log-odds ratio of the posterior distribution of the replacement mutation frequency compared to the expected distribution for the germline sequence, to quantify the strength of selection (117); the integration of phylogenetic information (118, 119); and estimation of the null distribution for the number of replacement mutations so that selection effects can be calculated for a single sequence (120).

While there is general agreement that purifying selection typically acts on FWRs, reports have been inconsistent as to whether diversifying selection acting on CDRs can (94, 115, 117) or cannot (114, 121) be detected at the repertoire level. Meanwhile, a review of available structural data found no relation between hot spot motifs and observed substitutions; the latter were instead strongly correlated with antigen contacts and contributions to calculated binding energy (122). In addition, a recent study found that the need to distinguish between closely related foreign and self antigens can drive the expansion of higher affinity clonal variants that remain subdominant in the absence of self antigen (123), demonstrating another way in which selection can influence the observed substitutions in a repertiore. On the other hand, an in-depth analysis of an antibody against influenza hemagglutinin found that mutability and selection synergized, such that replacement mutations expected to occur more frequently under a neutral model were also more like to be selected once generated (124). It is, therefore, clear that more work is needed to resolve when the effects of selection must be explicitly accounted for and when they can be implicitly included by the use of substitution profiles or other similar abstractions. Structural and biophysical characterizations of common versus rare substitutions should help resolve this question and will also be important for understanding the underlying biological mechanisms.

## Vaccine Implications

Reverse vaccinology 2.0 (125, 126) is a strategy for rational vaccine design that starts by characterizing the epitope targeted by an effective natural antibody and selecting or designing an immunogen that can elicit a similar antibody in other individuals. One particular implementation is lineage-based vaccine design, which attempts to find a series of immunogens that can together induce a vaccine-elicited antibody to recapitulate the ontogeny of a known lineage (127–130). Both strategies rest on the assumption that antibody elicitation is fundamentally reproducible. Thus, lineage-based vaccine design for HIV has focused on “classes” of antibodies (128) with similar genetic characteristics that have been observed in multiple donors. Despite genetic and structural similarity, however, several obstacles to the successful design of a vaccine capable of eliciting protective classes of antibodies remain to be overcome.

In particular, antibodies capable of broad neutralization of HIV have particularly high levels of SHM (128, 131, 132) and tend to be enriched for rare substitutions (104, 133, 134) (Figure 1). Extraordinary levels of SHM (15–35% nucleotide mutations) are characteristic of antibodies targeting HIV (135), and elevated levels of SHM have also been observed in other types of chronic infection and in systemic autoimmune disorders (136). By contrast, the maximum level of SHM that has been observed in vaccine-responsive antibodies is 8–10% nucleotide mutations, even after multiple doses (137, 138).

Fortunately, however, many mutations found in broadly neutralizing antibodies (bnAbs) against HIV appear to be unnecessary for full function (139, 140). In fact, two HIV bnAbs have recently been reported with at least 50% breadth and less than 10% nucleotide mutation in V_H_: CAP256-VRC26.25 (141) and DH270.1 (142). Importantly, though, both contain other unusual features. CAP256-VRC26.25 has an extraordinarily long heavy chain CDR3 of 38 amino acids, including a 1 amino acid insertion relative to the inferred naive ancestor (141), while the neutralization activity of DH270.1 depends on a critical Gly64Arg (IMGT numbering) mutation in a canonical SYC cold spot (142). As noted above, such rare mutations are generally enriched in HIV bnAbs compared to flu bnAbs (Figure 1) and antibodies from normal repertoires or induced by a vaccine (104). While accumulation of some rare substitutions may be incidental to the overall level of SHM (33, 104), a recent report demonstrated that half of the HIV bnAbs studied have accumulated significantly more rare mutations than expected under a neutral evolutionary model of SHM (104). Similarly, several positions with low intrinsic mutation rates were determined to be significantly enriched in a class of V_H_1-2-derived HIV bnAbs, based on their recurrence in members of that class (133). These observations suggest that, as for the DH270 lineage, at least some rare mutations may be functionally important. Indeed, this has recently been confirmed for three additional HIV bnAbs (104). The identification of critical rare mutations and strategies to reproduce them will be central to the success of lineage-based vaccine design.

One possible approach is to design immunogens capable of exerting strong selection on rare mutations as soon as they occur (104). However, even mutations that increase the affinity of an antibody 10-fold take much longer to dominate a germinal center reaction than would be expected from a simple model of SHM (91, 143). Moreover, recent work has shown that lower affinity subclones can persist in germinal centers (98–100), which may prevent antibodies with the desired rare substitution from reaching protective levels, even with an optimal immunogen. Indeed, while several recent studies in transgenic mice have elicited B cells enriched for substitutions present in the targeted mature antibody (144–146), none have yet specifically elicited critical rare substitutions or fully recapitulated the neturalization activity of the target antibodies. Notably, however, the most successful example focuses on PGT121 (146), which contains fewer rare substitutions than many other HIV antibodies (104, 134). It may, therefore, be more prudent to choose lineage-based vaccine design targets by avoiding those with functionally important rare substitutions (33, 134).

## Conclusion

The mechanisms of antibody diversification have evolved to achieve a balance between the plasticity needed to successfully bind to unknown novel antigens and the robustness needed to do so in a biologically feasible manner. This results in a series of patterns and variations that can be studied computationally both to illuminate the underlying cellular processes and to predict the response to specific manipulations. As advances in technology have made it possible to collect ever larger datasets, our ability to detect and understand these patterns has grown, as well. The insights provided thus far by substitution profiles and related concepts have already begun to be applied to antibody engineering and vaccine design. Concurrently, work is ongoing to understand the biology behind these patterns and to develop them into predictive models of immune function.

## Author Contributions

CS wrote the paper. All authors reviewed, commented on, and approved the manuscript.

## Conflict of Interest Statement

The authors declare that the research was conducted in the absence of any commercial or financial relationships that could be construed as a potential conflict of interest.
